# Risks and Preventive Strategies for *Clostridioides difficile* Transmission to Household or Community Contacts during Transition in Healthcare Settings

**DOI:** 10.3201/eid2707.200209

**Published:** 2021-07

**Authors:** Ramin Asgary, Jessica A. Snead, Nabeel A. Wahid, Vicky Ro, Marina Halim, Judy C. Stribling

**Affiliations:** George Washington University, Washington, DC, USA (R. Asgary);; Weill Cornell Medical College of Cornell University, New York, New York, USA (R. Asgary, N.A. Wahid, J.C. Stribling);; New York Presbyterian Hospital of Cornell University, New York (J.A. Snead, N.A. Wahid, M. Halim);; Columbia University Vagelos College of Physicians and Surgeons, New York (V. Ro)

**Keywords:** Clostridioides difficile, Clostridioides difficile infection, CDI, bacteria, infection, systematic review, transmission, household, community, healthcare settings, transition, risks, preventive strategies, household contacts, community contacts

## Abstract

The burden of *Clostridioides difficile* infection (CDI) has greatly increased. We evaluated the risks for CDI transmission to community members after hospitalized patients are discharged. We conducted a systematic literature review in MEDLINE/PubMed, EMBASE, CINAHL plus EBSCO, Web of Science, Cochrane Library, and gray literature during January 2000‒February 2019 and identified 4,798 citations were identified. We eliminated 4,554 citations through title and abstract screening; 217 additional citations did not meet full criteria. We reviewed texts for the 27 remaining articles qualitatively for internal/external validity. A few identified studies describing risks to community members lacked accurate risk measurement or preventative strategies. Primary data are needed to assess efficacy of and inform current expertise-driven CDI prevention practices. Raising awareness among providers and researchers, conducting clinical and health services research, linking up integrated monitoring and evaluation processes at hospitals and outpatient settings, and developing and integrating CDI surveillance systems are warranted.

*Clostridioides* (the genus name of this bacterium was changed from *Clostridium* to *Clostridioides* during 2018) *difficile* infection (CDI) is responsible for almost half a million infections and ≈29,000 deaths in the United States annually (*1*). During 2000‒2014, the number of hospitalizations from CDI increased from 134,518 to 361,945, and the financial contribution to inpatient healthcare expenditure increased from $0.5 billion to $3.9 billion (*2*). Risk factors for CDI and colonization include older age, recent hospitalization, recent use of antimicrobial drugs, and use of proton-pump inhibitors (*3*). Transmission of *C. difficile* occurs through the spread of spores primarily through environmental contamination, hands of healthcare personnel, and asymptomatic carriers (*4*). Several well-established guidelines recommend strategies in the inpatient setting to prevent and treat CDI. Prevention methods strongly recommended in the guidelines within an acute-care setting include isolating patients with CDI in private rooms with private toilets, using gloves and gowns when entering rooms with CDI patients, using soap and water when entering or exiting a CDI patient room, and cleaning reusable equipment with a sporicidal disinfectant (*4*). For treatment, the 2017 update by the Infectious Diseases Society of America (IDSA) and the Society for Healthcare Epidemiology of America (SHEA) recommends stopping causing antimicrobial drugs and using oral vancomycin or fidaxomicin, or intravenous metronidazole as a less preferred alternative, in most cases of CDI (*4*).

Although classically believed to be a hospital-acquired infection, *C. difficile* has also proven to be a major community pathogen. Although the 2017 IDSA/SHEA update recognizes the role of CDI in the community, it gives no specific prevention strategies to use at home (*4*). Community-acquired *C. difficile* might account for more than one third of total CDI cases, and patients tend to be younger and have less recent exposure to antimicrobial drugs and less exposure to healthcare settings than other persons who have CDI (*5**,**6*).

Because many patients hospitalized for CDI are discharged before completing full-course treatment or complete resolution of diarrhea, a common conundrum is deciding what prevention strategies are effective to be recommended at home after discharge to prevent the spread of infection to household or community contacts. Although substantial data and consensus guidelines exist for effective prevention strategies in the inpatient setting, similar data appear more sparse in the community setting. In this study, we systematically assessed data regarding the rate and role of the spread of *C. difficile* from an index hospitalized patient to the patient’s household members and community contacts. We also aimed to identify potential effective preventive strategies within the community.

## Methods

For this study, we defined the population of interest as patients who had positive test results for CDI and who had another household member or contact with a patient who had been previously given a diagnosis of and treatment for *C. difficile* diarrhea. We defined a positive test result for CDI as a patient who had diarrhea sample that had positive results in a glutamate dehydrogenase antigen test, both toxin A and B tests, or a nucleic acid amplification test in the setting of either negative glutamate dehydrogenase test result or toxin A and B test results, or positive stool culture, regardless of diarrhea symptoms (i.e., active CDI vs. asymptomatic carrier).

### Data Sources and Searches

We conducted a systematic review of literature in the databases MEDLINE, EMBASE, CINAHL plus EBSCO, Web of Science, PubMed, and The Cochrane Library, as well as gray literature, including abstracts/proceeding of gastroenterology, infectious disease, and related professional societies annual meeting, and guidelines by professional associations, all published during January 1, 2000‒February 19, 2019. In addition to the primary literature search, we performed a snowballing method and checked references cited in current guidelines and the most relevant articles from our search. We developed a list of key search terms (Table 1) during multiple brainstorming sessions (involving clinicians, contributors, and a specialized librarian) and through an extensive review of Medical Subject Headings (MeSH) terms from relevant articles identified through preliminary searches in PubMed. We divided the search terms into 2 search buckets, 1 centered around “*Clostridium difficile*” (all related MeSH terms and possible text words) and 1 centered around “carrier state” and “cross infection” (all related MeSH terms and possible text words). Furthermore, we used the OVID Medline strategy (Appendix) to search all databases by using appropriate thesaurus terms and natural language. The study was registered at the PROSPERO Registry as no. CRD42019118021 (study protocol provided in the Appendix).

**Table 1:** Search terms and databases used for systematic review of *Clostridioides difficile* infection*

Bibliographic database	Search terms/condition	Search terms/carrier state
OVID MEDLINE	*Clostridium difficile*, *Clostridium* Infections, *Clostridium* adj4 poisoning, *Clostridium* Perfringen, *Clostridium* sordell*, Infect* adj3 perfringen*	Carrier State, carrier and state, Cross infection, Cross and Infect*, infect* adj2 nosocomial
EMBASE	*Clostridium difficile*, *Clostridium* difficills*, *Clostridium* Infection, Clostridial Disease, Clostridial Infection* Clostridi*adj4 poisonin*, Clostridi* perfringen*, Clostridi* Sordell*	Carrier State, Cross Infection, Infect* and Cross, Infect* adj2 nosocomial
Web of Science	*Clostridium difficile*, *Clostridium* Infections, *Clostridium* Infection, *Clostridium* Poisoning, *Clostridium perfringens*, *Clostridicum* Perfringen, *Clostridium* Sordellii	Carrier State, Cross Infection, Nosocomial Infection, Nosocomial Infections
Cochrane Library	*Clostridium difficile*, *Clostridium* Infections, *Clostridium *Poisoning, *Clostridium* *perfringens*, *Clostridium sordellii*	Carrier State, Cross Infection, Nosocomial Infection, Nosocomial Infections
Gray literature	*Clostridium* Infection	Cross Infection

*Filters/limits were limit 2000‒present and English language. Asterisks indicate truncation for other variations or modified versions of a searched word (e.g., shortened, misspelled, or differently spelled versions). This search includes all versions of *Clostridi *and *Clostridium* *difficile*, including *Clostridioides*, which also are shown in citations used for this paper.

Inclusion criteria were studies that defined laboratory testing for *C. difficile* detection or used and measured diarrheal episodes or used any test to detect infection; measured or included a contact or an exposure with patients previously given a diagnosis of *C. difficile* diarrhea in hospital settings; measured outcomes among outpatient or community persons who were exposed in the form of rates or number of events; and mentioned or described an actual intervention (treatment such as antimicrobial drugs for the CDI index case, which is hypothesized to decrease the period of infectiousness and subsequent transmission or a prevention strategy, such as handwashing and surface cleaning with sporidicial antiseptic and contact isolation). Exclusion criteria were nonhuman studies, studies not published in English, studies that did not specifically describe the study population, and studies that did not describe any form of CDI infection or did not reference any treatment or prevention strategy.

### Data Extraction, Quality Assessment, and Data Synthesis and Analysis

We designed a 4-stage screening process to select the most relevant literature for review. First, we developed search terms along with a search algorithm and searched databases for articles containing the key search terms in their title or abstract. Second, we reviewed the titles and abstracts of these articles for exclusion and inclusion criteria. We examined whether a study had an index patient who had diarrhea caused by CDI in the hospital, an exposure that existed outside the hospital, an outcome after that exposure measured with laboratory tests or clinical diagnosis, and an intervention (either preventive or therapeutic) that was applied to the index patient or other exposed persons to protect against subsequent CDI at the community level. If there was no intervention, we set to record the rates of postexposure infection among contacts. Third, we qualitatively reviewed the full texts of the remaining articles that had not been excluded to confirm that they met the inclusion and exclusion criteria and to assess them for their sample size, outcome measures, biases, comparison of rates and outcomes, efficacy of their treatment or preventive measures, and internal/external validity.

We also applied a snowballing method by reviewing references and citations to current guidelines and panel of expert recommendations selected for the full text review. At the end of this process, we retrospectively read through all 217 articles from phase 2, even though they did not fully meet the inclusion criteria, to ensure that all potential relevant information was captured. We then organized and reported findings according to the Preferred Reporting Items for Systematic Reviews and Meta-Analysis guidelines (*7*). A dedicated librarian with expertise in conducting systematic review performed the database search and imported search results into Covidence software (https://www.covidence.org) for review. Two reviewers screened the articles from stages 1 and 2 independently. Another tie-breaker reviewer looked at the articles that were discordant. The texts of remaining articles were reviewed by >2 reviewers.

## Results

We found 4,798 articles through our search strategy. We compiled more detailed descriptions of search hits from specific databases (Table 2). After applying the exclusion criteria, we eliminated 4,554 articles through title and abstract screening. We screened the abstracts for the remaining 244 articles for inclusion criteria; we eliminated 217 of those for not meeting the full criteria. The full text of the remaining 27 articles were read to confirm eligibility (Figure).

**Table 2:** Results of literature review search on prevention and treatment/management of *Clostridioides difficile* infection to family members and community from an index hospital patient, by database, September 2019

Database	Search strategy for prevention	Search strategy for management	Results	Key features of search engine
PubMed	Full search	Full search	2,215	Index to articles in medical journals and other selected biomedical literature
Cochrane Library	Full search	Full search	435	Database of systematic reviews of primary research in human healthcare and health policy
Web of Science	Search limited to 50 terms	Search limited to 50 terms	1,494	Helpful for topics that border on social science
EMBASE	Full search	Full search	1,653	European alternative to PubMed; helpful for topics with an international focus
Gray literature	Full search	Full search	1	Manifold document types produced on all levels of government, academics, business and industry in print and electronic formats.

**Figure:**
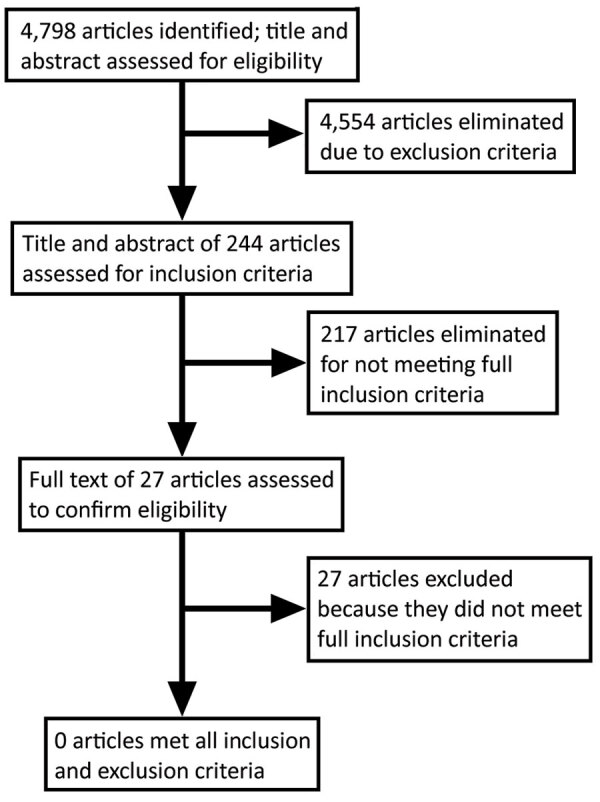
Process of selecting studies suitable for inclusion in the final review of the literature on prevention and treatment/management of *Clostridioides difficile* infection to family members and community from an index hospital patient, by database, September 2019.

None of the articles evaluated transmission of *C. difficile* from an infected person in the hospital to someone in the community, long-term acute care facility, nursing home, or subacute rehabilitation center. Some common reasons for eliminating articles included nonclinical studies that used computer modeling to study transmission, studies that only included exposure occurring within instead of outside the hospital, and studies that had no interventions described to prevent transmission. For example, one study interviewed 1,013 patients who had confirmed community-acquired *C. difficile* and showed that 11 patients had a household member with active CDI (*6*). Of patients with community-acquired CDI and no outpatient healthcare exposure, the odds ratio of having a household member with active CDI was 6.8 (95% CI 0.7–65.9) compared with patients who had high-level outpatient healthcare exposure (*6*). However, the study did not verify the infectious status of the index patient and did not examine what was performed to prevent transmission to household contacts.

Another study examined household transmission for 2,222 patients who had confirmed *C. difficile* infection diagnosed at the Centre Hospitalier Universitaire de Sherbooke (Sherbooke, Quebec, Canada). The study identified 1,061 spouses and 501 children <25 years of age living with the index patients (*8*). Five spouses and 3 children developed *C. difficile* infection within a year after discharge of the index patients (attack rate 4.71 cases/1,000 persons for spouses and 5.99 cases/1,000 persons for children of index cases). Similar to the study of Chitnis et al. (*6*), this study did not define what isolation or prophylactic measures were taken to reduce transmission to household contacts. However, a more recent study by Miller et al. conducted among 194,424 enrollees, published after our original search was completed, indicated a 12.47 incidence rate ratio among household contacts of DCI patients discharged from tertiary care centers (*9*). In addition, Loo et al. evaluated probable transmission rates of 1.5% and possible transmission rates of 7.5% for household contacts of 51 CDI patients (*10*).

Some studies reported community-acquired CDI, but did not explicitly report an exposure to hospitalized patients who had *C. difficile* infection (*11**,**12*). For example, Bloomfield and Riley reported estimated rates of community acquired *C. difficile* infection in North America ranging from 20% to 32% (*12*). This study also showed that nonhuman reservoirs, including animals and food, have shown positive results for *C. difficile* infection. However, these findings have yet to be replicated by additional studies. Another source of community-associated *C. difficile* infection studied was healthcare exposure. For example, Chitnis et al. (*6*) showed that 82% of their patients had some exposure to healthcare within 12 weeks before infection, including outpatient dental or physician office visits and dialysis. They also showed known traditional risk factors: 64% used antimicrobial drugs within 12 weeks before infection, and 27.7% used proton pump inhibitors (Table 3).

**Table 3:** Results of qualitative assessment of studies evaluating risk for CDI in the community*

Study (reference)	Study type	Year	No. persons/studies	Setting	Actual risk/assumed risk	Intervention
Pépin et al. (*8*)	Retrospective	2012	2,222 *C. difficile* patients	Household contacts	Children attack rate: 4.71 cases/1,000 persons; spouse attack rate: 5.99 cases/1,000 persons	None
Chitnis et al. (*6*)	Retrospective and telephone interview	2013	984 community- acquired *C. difficile* patients	Household contacts	Odds of community- acquired CDI if no outpatient healthcare exposure: 6.8 (95% CI 0.7–65.9); odds of community-acquired CDI if low level outpatient healthcare exposure: 6.9 (95% CI 0.9‒56.7)	None
Durovic et al. (*11*)	Narrative review	2018	24 studies	Other healthcare facilities and community	Not measured	None
Bloomfield and Riley (*12*)	Narrative review	2016	NA	Household contacts	Estimated rate of community acquired CDI in North America: 20%–32%	None
Loo et al. (*10*)	Prospective	2016	51	Household contacts	Probable transmission: 1.5%; possible transmission: 7.5%	None, but type of soap for handwashing was recorded
Miller et al. (*9*)	Case‒control	2020	194,424 enrollees	Household contacts	IRR 12.47 (95% CI 8.86–16.97)	None

*CDI, *Clostridioides difficile* infection; IRR, incidence rate ratio.

We identified consensus articles from organizations, such as the International Infection Control Council, IDSA, and SHEA. In addition, the American Nursing Association endorses the approach of the Centers for Disease Control and Prevention, which in return endorses IDSA guidelines. Many recommendations were guided by expert opinion, rather than primary research on CDI transmission from the hospital setting to the community. Although many of the guidelines are not guided by primary research results, we highlighted some current inpatient practices for treating and preventing transmission of CDI in the inpatient setting (Table 4).

**Table 4:** Summary of current CDI prevention and treatment guidelines for in-patient settings*

Society/reference	Scope	Prevention recommendations	Treatment recommendations
Infectious Disease Society of America and Society for Healthcare Epidemiology of America 2017 Update (*4*)	Primarily targeted toward pediatric and adult inpatient facilities	Isolate patients in private rooms with single toilets. In resource limited settings, prioritize incontinent patients in private rooms; cohort patients with same organism(s) when necessary. Healthcare workers should use gloves and gowns. Hand hygiene: use soap and water or alcohol-based hand hygiene; prefer handwashing in hyperendemic areas. Initiate isolation preemptively; continue isolation for at least 48 h after diarrhea resolves or until discharge. Encourage patient had washing and showering. Use disposable equipment when possible and disinfect with sporicidal disinfectant. Daily cleaning with sporicidal agent should be considered. Insufficient data for isolating asymptomatic carriers. Minimize frequency and duration of antimicrobial drugs; initiate antimicrobial rug stewardship programs.	Discontinue inciting antimicrobial drugs; start treatment empirically when laboratory delay or fulminant CDI. Initial episode: oral vancomycin or fidaxomicin; Metronidazole second line. Fulminant CDI: prefer oral vancomycin (rectally if ileus). Recurrent CDI: oral vancomycin with taper OR 10-d course of fidaxomicin OR 10-d course of vancomycin if metronidazole was used for previous episode. Fecal transplantation recommended in patients with multiple recurrences who failed appropriate antimicrobial drugs. Above guidelines for adults
American College of Gastroenterology 2013 (*13*)	Primarily targeted toward adults in acute care facilities	Antimicrobial drug stewardship reduces risk for CDI. Isolate CDI patients in private or in a room with another CDI patient for at least 48 h after diarrhea stops. Encourage hand hygiene and barrier protection (gloves and gowns). Preferentially use single-use equipment; other equipment should be cleaned thoroughly with Environmental Protection Agency‒registered *C. difficile*‒-sporicidal label claim or 5,000 ppm chlorine-containing agents.	Stop inciting antimicrobial drugs if possible. Mild-to-moderate CDI: metronidazole for 10 d. Severe: CDI: oral vancomycin for 10 d. Severe and complicated CDI: oral vancomycin plus intravenous metronidazole. Surgical consultation should be obtained for all patients with complicated CDI. If no response to metronidazole for 5–7 d, change to vancomycin. Rectal vancomycin if oral antimicrobial drugs cannot reach a segment of the colon. CT abdomen pelvis recommended in patients with complicated *C. difficile* . Recurrent CDI: first recurrence, same regimen used previously; second recurrence, pulsed vancomycin; third recurrence, fecal microbiota transplant should be considered
European Society of Clinical Microbiology and Infectious Diseases (*14**,**15*)	Primarily targeted toward adults in acute care facilities in endemic and outbreak settings	No specific recommendations regarding most effective technique for handwashing. Prefer handwashing over alcohol-based hand rub in outbreak settings, but not in endemic settings. Use gloves, gowns/disposable aprons to decrease transmission. Use daily sporicidal disinfection of rooms. No-touch disinfection systems may be effective in reducing transmission. Restrict antimicrobial drug agents/classes and decrease duration to decrease rates of CDI. Provide education to healthcare workers on prevention strategies.	Nonsevere CDI in nonepidemic situations: can consider stopping inducing antimicrobial drugs and observing clinical response for 48 h before starting therapy; first-line treatments: oral metronidazole for 10 d, oral vancomycin for 10 d, oral fidaxomicin for 10 d. Severe CDI: prefer vancomycin over metronidazole; fidaxomicin noninferior to vancomycin. Total abdominal colectomy in following cases: perforation of colon or systemic inflammation and deterioration of clinical condition despite maximal antimicrobial drugs. First recurrence and multiple recurrences (nonsevere): prefer oral vancomycin or oral fidaxomicin. When oral treatment is unavailable, intravenous metronidazole is recommended.
World Society of Emergency Surgery 2019 (*16*)	Primarily targeted toward adults in acute care facilities	Use proper antimicrobial drug stewardship. Place *C. difficile* carriers (in addition to those actively effected) on contact precautions. Hand hygiene with soap and water.	Stop unnecessary antimicrobial drugs and proton-pump inhibitors. Do not start empiric treatment for CDI unless there is strong clinical suspicion. Oral metronidazole limited to treatment for initial episode of mild-moderate CDI; use oral vancomycin if refractory to metronidazole. Severe CDI: use vancomycin or fidaxomicin. First recurrence: use vancomycin or fidaxomicin. Multiple recurrences: oral vancomycin using a tapered or pulsed regimen. Consider fecal microbiota transplantation if multiple recurrences with failure of appropriate antimicrobial drug treatments. Use vancomycin enema if oral antimicrobial drugs cannot reach colon. Indications for surgery (patients with severe CDI who progress to systemic toxicity should undergo early surgical consultation; Fulminant colitis: consider resection of entire colon). Consider prophylactic probiotics in inpatients receiving antimicrobial drugs during high-risk period before disease develops. Probiotics might be effective in the prevention of recurrent CDI in conjunction with standard antimicrobial drugs. Can consider monoclonal antibodies (bezlotoxumab) to prevent recurrences of CDI in patients with 027 epidemic strain in immunocompromised patients and in patients with severe CDI. Intravenous immunoglobulin should only be used as adjuvant therapy in patients with multiple recurrent or fulminant CDI.

*CDI, *Clostridioides difficile* infection.

## Discussion

Increasingly, the extent and role of hospital-acquired infections, excessive use of antimicrobial drugs, drug-resistant bacterial infections, and decreased efficacy of common and available antimicrobial drugs as a serious threat to individual and population health, and health agencies in the United States and elsewhere have called for measures to address these factors (*17*). *C. difficile* continues to be among the highest burden of hospital-acquired infections, such that IDSA, SHEA (*4*), the American College of Gastroenterology (*13*), and the European Society of Clinical Microbiology and Infectious Diseases (*14*,*15*), have all published guidelines for preventing and managing *C. difficile* in inpatient settings. Available data demonstrate the considerable extent of *C. difficile* in the community (*12*), evidence of *C. difficile* on household surfaces among patients who have recurrent CDI (*18*), a rate of probable transmission of 1.5% and a rate of possible transmission of 7.5% for household contacts of discharged CDI patients (*10*). More recently, the incidence rate ratio of CDI was reported as 12.47 for household contacts of discharged patients who have CDI (*9*). However, no systematic data provide evidence of effective prevention strategies at the community level and with household contacts of index patients discharged from the hospital. Consequently, practitioners often do not provide specific prevention recommendations for CDI to patients or family members outside the hospital. Consequently, practitioners often do not provide specific prevention recommendations for CDI to patients or family members outside the hospital.

In this systematic review, we applied a comprehensive search strategy in a variety of search engines to cover complementary areas of the literature relevant to CDI prevention and treatment, including the gray literature and data from related professional associations. Through this extensive search, we were not able to find any publications that evaluated strategies to prevent or manage CDI among contact family or community members of an index patient. Therefore, we state that no data are currently available to demonstrate whether the prevention and management strategies that are widely used and included in proposed guidelines for inpatient or hospital setting are efficacious, feasible, or effective to prevent transmission outside the hospital.

The reasons for this lack of data are likely multifactorial. A fragmented healthcare system does not provide opportunities to identify and record outpatient episodes and related illnesses associated with inpatient CDI diagnosis. In addition, no systematic approach has been established to collect data at the patient level through providers, and no public health tracing or follow-up process with family members exists. Departments of health at the state level do not routinely collect data related to CDI patients or subsequent infections (*19*). The providers caring for index or subsequently exposed patients often lack the instruction or support necessary for evaluating patients after hospital discharge and their family or community contacts. There might be low rates of secondary symptomatic infections in the household setting. Furthermore, there is probably a lack of recognition of the burden of CDI among outpatient health providers, and laboratory report systems are not in place to send reminders. Potential consequences of this lack of strong data include inadvertent transmission of CDI from the community back to the healthcare environment, increased financial cost to health system from treating preventable cases of secondary CDI, and probably an increasing number of multidrug-resistant CDI. The Institute of Medicine has emphasized the burden of hospital-acquired infections and the need for systematic approaches and delineated framework and processes for moving forward (*20*,*21*).

We have provided a summary of current practices in the inpatient settings because we realize that in the absence of primary data, the recommended approaches need to include all levels of evidence to direct the actual practice. Nevertheless, the role of primary approaches, such as antimicrobial drug stewardship, could not be overemphasized. Furthermore, we suggest that a range of overarching initiatives is needed to address the risk and subsequent burden of transmission of CDI to the community. Perhaps the most useful area to focus on is the development of a monitoring and evaluation process in the hospital setting that can ensure that relevant data are available to outpatient providers at the time of discharging the index patient. Proper data collection processes should be added into the current system of collecting and monitoring health data by developing tools and reinforcing accurate documentation and tracking of CDI cases and their sequelae. A direct link between providers in the outpatient and hospital settings to identify and address subsequent CDI should not be overlooked. Simple strategies, such as follow-up telephone calls and gathering information from family members, could help determine the possibility or the extent of the disease at the patient level through similar initiatives commonly used for postsurgical interventions (*22**–**27*).

There is also a need for direct primary research on the feasibility and efficacy of specific CDI prevention and management strategies after hospital discharge. Prevalence studies evaluating outcomes at individual and household levels, and interventional cohorts, including different types of preventive or management strategies for CDI should be considered because they are likely to provide useful data.

We did not include studies published in languages other than English. However, our preliminary search did not identify this limitation as a major gap in evidence. Data regarding the efficacy of prevention strategies at the community level might exit in the form of reports and proposals developed in departments of health in or outside the United States that were not captured in our extensive systematic review. 

Our systematic review indicates a need for research that evaluates the efficacy and effectiveness of various CDI prevention and management strategies after infected patients are discharged from in-patient settings. Ultimately, this research will enable the field of CDI and multidrug-resistant infections to transition from one that is largely extrapolative and expertise driven to one that is more evidence based. The current guidelines do not give any recommendations on how to prevent and manage CDI among family members and community contacts after hospital discharge of an index patient. However, guidelines do recommend assessment and monitoring, clearly emphasizing the need for good data and evidence. There are clearly challenges at the research and practice level that need to be systematically addressed. To start, perhaps there is a need to appropriately raise awareness of the problem among clinical providers and researchers. Concurrently, conducting related clinical and population level research, setting up and connecting monitoring and evaluation programs at hospital and outpatient settings, and developing CDI-related data within public health surveillance are warranted.

Appendix Additional information on risks and preventive strategies for *Clostridioides difficile* transmission to household or community contacts during transition in healthcare settings.
