# Supplementary material for: Risks and Preventive Strategies for Clostridioides difficile Transmission to Household or Community Contacts during Transition in Healthcare Settings
Source: Emerg Infect Dis. 2021 Jul;27(7):1776–82. doi: 10.3201/eid2707.200209 (PMC8237889; doi:10.3201/eid2707.200209)
Supplement: Appendix — Additional information on risks and preventive strategies for Clostridioides difficile transmission to household or community contacts during transition in healthcare settings. [file 20-0209-Techapp-s1.pdf]

Article DOI: https://doi.org/10.3201/eid2707.200209

Risks and Preventive Strategies for *Clostridioides difficile* Transmission to Household or Community Contacts during Transition in Healthcare Settings

Appendix

PROSPERO Proposal

Table of Contents

Requirements

1. Review title
2. Original language title
3. Anticipated or actual start date
4. Anticipated completion date
5. Stage of review at time of this submission
6. Named contact
7. Named contact email
8. Named contact address
9. Named contact telephone number
10. Organizational affiliation of the review
11. Review team members and their organizational affiliations
12. Funding sources/sponsors
13. Conflicts of interest
14. Collaborators

15. Review question

16. Searches

17. URL to search strategy

18. Condition or domain being studied

19. Participants/population

20. Intervention(s), exposure(s)

21. Comparator(s)/control

22. Types of study to be included

23. Context

24. Main outcome(s)

25. Additional outcome(s)

26. Data extraction (selection and coding)

27. Risk for bias (quality) assessment

28. Strategy for data synthesis

29. Analysis of subgroups or subsets

30. Type and method of review

31. Language

32. Country

33. Other registration details

34. Reference and/or URL for published protocol

35. Dissemination plans

36. Keywords

37. Details of any existing review of the same topic by the same authors

38. Current review status

39. Any additional information

40. Details of final report/publications(s)

Proposal for Study

Review Title

Systematic review of *C. difficile* transmission to household contacts during transition in healthcare settings: the rate and significance of transmission

Original language title

English

Anticipated or actual start date

November 26, 2018

Anticipated completion date

June 1, 2019

Stage of review at time of this submission

Literature search

Named contact

Ramin Asgary, MD, MPH

Named contact email

raa9102@med.cornell.edu

Named contact address

525 East 68th

Box 331

New York, New York 10065

212–746–5924

Organizational affiliation of the review

Weill Cornell Medicine

Review team members and their organizational affiliations

Jessica Snead, PharmD, Principle Co-Investigator, [New-York Presbyterian Hospital], Marina Halim, Research Assistant [Arnold and Marie Schwartz College of Pharmacy, Long Island University], Judy Stribling [Weill Cornell Medicine], Ramin Asgary, MD, MPH, MSc, Principle Investigator, [Weill Cornell College of Medicine]

Funding sources/sponsors

None

Conflicts of interest

None

Collaborators

None

Review question

What is the rate and significance of transmission of *Clostridium difficile* from the index case of patients treated for *C. difficile* diarrhea to the patients' household members and contacts?

Searches

MEDLINE, EMBASE, CINAHL plus EBSCO, Web of Science, PubMed, and The Cochrane Library (Cochrane Database of Systematic Reviews), Psychinfo, Google Scholar and gray literature including abstracts/proceeding of Gastroenterology, infectious disease and related professional societies annual meetings, as well as guidelines by professional associations using only the English language from January 2000 and beyond.

URL to search strategy

?

Condition or domain being studied

Clostridium difficile

Participants/population, intervention(s), exposure(s)

Inclusion Criteria

The population of interest would be patients who test positive for *Clostridium difficile* infection in the setting of another household member or contact having previously been given a diagnosis of and treatment for *Clostridium difficile* diarrhea. A positive test of *C. difficile* infection would be defined as patients with diarrhea testing positive for GDH antigen test, both toxin A and B test, or NAAT in the setting of either negative GDH antigen test or toxin A and B test, regardless of symptom of diarrhea (i.e., active *Clostridium difficile* infection versus asymptomatic carrier).

Exclusion Criteria

Nonhuman studies. Nonclinical studies. Studies that do not specifically describe the study population. Studies not published in English.

Comparator(s)/control

Not applicable.

Types of study to be included

Nonclinical studies is excluded. All other studies including randomized, observational, case studies, case series, case reports, meta-analysis, and reviews.

Context

No restriction is planned on study settings

Main outcome(s)

Rate or incidence, and consequences of *Clostridium difficile* infection in household contact of a previously diagnosed and treated patient with *Clostridium difficile* infection.

Additional outcome(s)

Duration of time from and nature of exposure (household member with diagnosis and treatment for *Clostridium difficile* diarrhea) and positive test result for *Clostridium difficile* infection of patient

Patient characteristics of population of interest to determine risk profile and factors

Characteristics of exposure that increases risk for infection

Treatment shortcoming or characteristics of treatment or prevention strategies that increase or mitigate risk for infection

Data extraction (selection and coding)

Selection of Studies

Search results will be collated into a reference management software to establish a central database with duplicate records removed. Two reviewers will independently screen titles and abstracts for eligibility for selection. Full text of all eligible selections will be reviewed, with inclusion of articles agreed upon by both reviewers. Discrepancies in eligibility and selection will be resolved between both reviewers; a third-party reviewer will resolve any discrepancies that are not resolved by the former 2 reviewers.

Risk for bias (quality) assessment

The Cochrane Effective Practice and Organization of Care (EPOC) Review Group quality criteria and PRISMA methodology will be used to assess studies and minimize bias. A checklist for different relevant biases along with methods to ascertain them is developed and applied to all eligible studies.

Strategy for data synthesis

Both qualitative and quantitative methods will be used to analyze data. Qualitative and descriptive analysis will be used for all primary and secondary outcomes. Quantitative analysis is planned, when applicable and data available, for average rate/risks ascertainment/incidences. For efficacy of treatment for primary index patient or of risks, RevMan software will be used when data available and if applicable. Three research team members will be involved in analysis and majority vote will be used.

Analysis of subgroups or subsets

This is primarily a descriptive analysis. For primary, as well as secondary outcomes, when data available, subgroup analyses will include analyses of covariates and logistic regression for sex, age, duration of exposure, other characteristics of exposure for index case, household contact, and characteristics of initial index case’s treatment such as antibiotic choice and its route and its duration, and other preventive strategies.

Type and method of review

Systematic review

Language

English

Country

Any country

Other registration details

?

Reference and/or URL for published protocol

?

Dissemination plans

?

Keywords

Clostridium difficile (all related MeSH terms), community, household, patient, infection, transmission (exposure, contact), risk, rate, incidence, side effect, antibiotics (all MeSH terms), diarrhea and related mesh terms, treatment and related mesh term (fecal, transplant), prevention, infection control, duration and time (and related MeSH terms)

Details of any existing review of the same topic by the same authors

None

Current review status

Review completed

Any additional information

Details of final report/publications(s)

Search strategy for CDI in MedLine/OVID

OVID MedLine

1. Exp *Clostridium difficile*/

2. (clostridium difficile OR c diff).mp [mp = title, abstract, original title, name of substance word, subject heading word, floating sub-heading work, keyword heading word, protocol, supplementary concept word, rare disease supplementary concept word, unique identifier, synonyms]

3. Exp *Clostridium* Infections/

4. *Clostridicum* infect*.mp. [mp = title, abstract, original title, name of substance word, subject heading word, floating sub-heading word, keyword heading word, protocol supplementary concept word, rare disease supplementary concept word, unique identifier, synonyms]

5. (*clostridium* adj4 poisoning).mp. [mp = title, abstract, original title, name of substance word, subject heading word, floating sub-heading word, keyword heading word, protocol supplementary concept word, rare disease supplementary concept word, unique identifier, synonyms]

6. *Clostridium* perfringen*.mp [mp = title, abstract, original title, name of substance word, subject heading word, floating sub-heading word, key heading word, protocol supplementary concept word, rare disease supplementary concept word, unique identifier synonyms]

7. Clostridcum sordell*.mp. [mp = title, abstract, original title, name of substance word, subject heading word, floating sub-heading word, keyword heading word, protocol supplementary concept word, rare disease supplementary concept word, unique identifier, synonyms]

8. 9infect* adj3 sordell*).mp [mp = title, abstract, original title, name of substance word, subject heading word, floating sub-heading word, keyword heading word, protocol supplementary concept word, rare disease supplementary concept word, unique identifier, synonyms]

9. (infect* adj3 perfringen*).mp. [mp = title, abstract, original title, name of substance word, subject heading word, floating sub-heading word, keyword heading word, protocol

supplementary concept word, rare disease supplementary concept word, unique identifier, synonyms]

10. One OR 2 OR 3 OR 4 OR 5 OR 6 OR 7 OR 8 OR 9

11. Exp Carrier State/

12. ((carrier and state*) OR (state* and carrier)). mp [mp = title, abstract, original title, name of substance word, subject heading word, floating sub-heading word, keyword heading word, protocol supplementary concept word, unique identifier, synonyms]

13. Exp Cross Infection/

14. ((cross and infect*) or (infect* and cross)). mp. [mp = title, abstract, original title, name of substance word, subject heading word, floating sub-heading word, keyword heading word, protocol supplementary concept word, unique identifier, synonyms]

15. (infect* adj2 nosocomial). mp [mp = title, abstract, original title, name of substance word, subject heading word, floating sub-heading word, keyword heading word, protocol supplementary concept word, rare disease supplementary concept word, unique identifier, synonyms]

16. Eleven OR 12 OR 13 OR 14 OR 15

17. !0 AND 16

18. Limit 17 to yr = ”2000 –Current”

19. Limit 18 to English language
